# Peripheral nerve stimulation for pudendal neuralgia and other pelvic pain disorders: current advances

**DOI:** 10.3389/fruro.2023.1323444

**Published:** 2023-12-18

**Authors:** Natalija Kovacevic, Larry Sirls, Jason Gilleran, Kenneth Peters

**Affiliations:** William Beaumont Hospital, Corewell Health, Department of Urology, Royal Oak, MI, United States

**Keywords:** pudendal neuralgia, chronic pelvic pain, peripheral nerve neuromodulation, sacral neuromodulation, pudendal neuromodulation

## Abstract

Chronic pelvic pain conditions such as pudendal neuralgia pose significant treatment difficulty due to their elusive etiology and diverse symptomatology. Initially approved as a third or fourth-line treatment of non-obstructive urinary retention and fecal incontinence, neuromodulation has also proven effective for pelvic pain associated with urinary dysfunction. Recently, sacral and pudendal neuromodulation has demonstrated efficacy in managing a spectrum of chronic pelvic conditions including refractory pudendal neuralgia. The individualized approach of peripheral neuromodulation has opened new avenues for tailored medical interventions, extending its application to conditions such as pudendal neuralgia, post sling pain, and vulvodynia. New technologies leading to miniaturized neuromodulation devices such as Freedom^®^ stimulators (Curonix), allows us to implant leads and modulate nerves at precise pain targets. Further experience and research is needed to assess the impact of targeted neuromodulation on managing complex pelvic pain conditions.

## Introduction

Chronic pelvic pain (CPP) poses considerable clinical difficulty due to its elusive etiology, diverse symptomatology, and negative impact on patients’ quality of life (1). It encompasses various conditions including pudendal neuralgia, interstitial cystitis (IC), pelvic floor dysfunction, vulvodynia, and more (1–3). Diagnosing CPP is challenging due to its non-specific symptoms and no consensus exists regarding the most effective treatment method. Consequently, patients explore various treatment options, often exhausting all possibilities without achieving lasting pain management.

Neuromodulation has emerged as a potential treatment for CPP, stemming from its successful application in managing complex regional pain syndrome (CRPS). CRPS, which is also believed to have a neuropathic component, has shown success with various forms of neuromodulation, including spinal cord stimulation (2). Given the similarities between CRPS and CPP, neuromodulation has gained traction in the treatment of various pelvic pain conditions. The pudendal nerve, comprising S2-S4 nerve roots, innervates the majority of the pelvis, highlighting the potential success of neuromodulation of those roots in the management of pelvic pain.

The pudendal nerve provides efferent motor innervation to the external genitalia, perineum, rectum, and anus and carries afferent sensory information from the posterior labia majora and scrotum, labia minora, vestibule, lower fifth of the vaginal canal, and the clitoris and penis. Furthermore, it is involved with clitoral and penile erections and innervates the external urethral and external anal sphincters (4). As a result, damage or injury to the pudendal nerve can result in pain across the regions it innervates, as well as urinary and fecal dysfunction, and sexual dysfunction.

In an effort to manage refractory cases of pudendal neuralgia, neuromodulation techniques have been employed, consisting of stimulation of various neural targets. These targets include the spinal cord, dorsal root ganglia, sacral nerve roots, conus medullaris, and direct stimulation of the pudendal nerve (5–7).

In this review we provide an overview of the applications of neuromodulation, and specifically peripheral nerve stimulation, in the management of pudendal neuralgia and related pelvic pain disorders such as persistent genital arousal disorder, vulvodynia, and post sling pain.

## Methods

This review was conducted using Preferred Reporting Items for Systematic Reviews and Meta-Analyses (PRISMA) guidelines. Once eligibility criteria were determined by the authors, all observational studies assessing the role of neuromodulation in pudendal neuralgia and pelvic pain disorders were evaluated. If abstracts met the eligibility criteria, the full text articles were obtained and screened.

Key words used in the PubMed search included were “neuromodulation AND pudendal neuralgia”, “peripheral neuromodulation AND pudendal neuralgia”, “neuromodulation AND pelvic pain”, “peripheral neuromodulation AND pelvic pain”, “neuromodulation AND pudendal nerve”, “neuromodulation AND pain”, “Interstim and pudendal neuralgia”, “pudendal block and pudendal neuralgia”. In our literature search, we did not identify any sham-controlled studies.

## Pudendal neuralgia

Pudendal neuralgia refers to the presence of pain along the distribution of the pudendal nerve. Originally described by Boisson in 1966, pudendal neuralgia is characterized by persistent and often severe pelvic pain (8). The Nantes criteria were introduced in 2008 to provide a standardized diagnostic approach for pudendal neuralgia and consist of five key elements:

1. Pain located in the anatomical region of the pudendal nerve 2. Worsened by sitting 3. Absence of pain at night, 4. No sensory loss during clinical exam, and 5. Pain improved after pudendal nerve block (9). Exclusion criteria include: 1. Exclusive pain in the coccygeal, gluteal, hypogastric, or pubic regions 2. Pruritus 3. Exclusively paroxysmal pain, and 4. Abnormal imagining findings that explain the pelvic pain (9).

Various treatment modalities for pudendal neuralgia include opioid and non-opioid analgesics, tricyclic antidepressants, pelvic floor physical therapy (PFPT), pudendal nerve blocks, and, in refractory cases, more invasive options such as nerve entrapment surgery (10). However, effects are often temporary with some patients experiencing no relief from their symptoms despite all available treatment options. In such cases, neuromodulation may be considered as an alternative option.

A potential advantage with neuromodulation is the reduction of opioid usage observed in pudendal neuralgia patients treated with Interstim. A retrospective review of 21 patients with IC with CPP between 2000 and 2002 found that mean morphine dose equivalents decreased by 36% (from 81.6 to 52.0 mg/day) after sacral neuromodulation (11). Among the 18 patients who reported using opioids prior to neuromodulation, four individuals discontinued narcotic medications after surgery.

## Pudendal neuromodulation

Sacral neuromodulation (SNS) was developed in the 1980s and gained FDA approval in 1997 for the treatment of urge incontinence, urinary frequency, and non-obstructive urinary retention (12, 13). Over time, its application has been expanded to include treatment of fecal incontinence and pelvic pain in conjunction with urinary and/or fecal incontinence (14, 15). However, up to 25% of patients do not respond to SNS (16). Conventional SNS relies on the stimulation of the S3 nerve root while the pudendal nerve encompasses the S2, S3, S4 nerve roots. By enhancing afferent stimulation through direct stimulation of the S2, S3, S4 nerve roots, it is possible to improve patient outcomes. For patients who fail S3 neuromodulation stimulating the pudendal nerve (S2,3,4) directly may give better outcomes. An explanation for the lack of SNS response in certain patients may lie in the afferent mapping of the pudendal nerve. Huang et al. discovered that S3 accounted for only 35% of afferent sensory fibers, whereas S2 contributed 60% of afferent innervation (17). Therefore, stimulating additional nerve roots, as with the pudendal nerve, may further improve clinical outcomes in patients with pudendal neuralgia.

The gate control theory offers a potential explanation for how pudendal neuromodulation may work in alleviating pain (18). According to this theory, the dorsal horn of the spinal cord contains “gates” that control the transmission of nociceptive and non-nociceptive signals. When non-nociceptive input is activated, it closes the gates, preventing nociceptive input from reaching the brain, thus reducing the perception of pain. In the context of pudendal neuromodulation, it is hypothesized that stimulation of the pudendal nerve triggers non-nociceptive input, leading to the closure of these gates and diminishing transmission of painful stimuli.

Limited data is available regarding the use of pudendal neuromodulation (PNM) in the treatment of pudendal neuralgia. In a three-patient series by Carmel et al., women with pudendal neuralgia experienced at least 80% reduction in pain (19). These results were maintained at 24-month follow-up. A retrospective review of 84 patients with IC/painful bladder syndrome (PBS) or overactive bladder (OAB) who underwent PNM, 71.4% of patients had positive response (defined as >50% improvement in symptoms) (16). Additionally, among the patients who had previously failed SNS, 93.2% (41/44) responded positively to pudendal lead placement.

Another study of pudendal neuralgia with 19 patients used questionaries to evaluate patients’ response to PNM (20). All patients reported improvements in pain after initial implantation. In the follow-up survey, eight out of 10 patients expressed satisfaction with PNM, and eight out of nine patients noted that PNM was more beneficial than pudendal nerve block (one patient perceived them to be equally effective). Notably, among patients who had previously failed SNS, three fourths found PNM to be effective.

Crescenze et al. aimed to identify what factors determine if patients with concomitant pelvic pain and voiding dysfunction benefit from pudendal neuromodulation (21). Patients with voiding symptoms alone were excluded. They observed that of patients responding to a pudendal nerve block, 90% responded to PNM and had a permanent lead implantation for pelvic pain. The study demonstrated a strong correlation between pain relief achieved from pudendal nerve blocks and success achieved from PNM.

Pudendal neuromodulation has demonstrated effectiveness even in cases of prior pudendal nerve entrapment surgery (PNES). In a study involving 15 patients who had prior PNES, 64% of patients reported improved pain after PNM, with 80% of patients undergoing implantation of the IPG (22). More importantly, the operative time did not differ significantly between the PNES and non-PNES groups (48 minutes vs. 50 minutes). This indicates that the presence of scarring, which can occur after PNES, does not preclude success from PNM. It is worth noting that two patients had challenging lead placements. Additionally, six out of 15 patients required re-operation, with three patients undergoing explanation of the Interstim device.

Hunter et al., presented a case of a 36-year-old female with right-sided pudendal neuralgia and deep midline vaginal pain after right pudendal nerve injury during robotic hysterectomy for cervical cancer (23). The patient did not have significant pain relief from treatments including medications, S3/S4 nerve root blocks, and caudal epidurals. However, continuous infusion at the right S3 nerve root provided partial relief with persistent left-sided pain. Subsequently a trial of bilateral pudendal nerve stimulation at the S3 nerve root was performed, resulting in a 75% improvement in symptoms within five days. Patient had residual perineal pain indicating that stimulation of S4 nerve root may be beneficial. Ultimately, patient underwent permanent lead implant with four leads placed into the bilateral S3 and S4 nerve roots. At four-year follow up patient reported two out of ten pain.

These findings show that pudendal neuromodulation is a promising treatment for pudendal neuralgia, with a notable number of patients experiencing pain relief and improved outcomes compared to SNS and nerve blocks. However, further studies are necessary to establish the efficacy and long-term outcomes of PNM in larger patient population.

## Sexual dysfunction and neuromodulation

It is hypothesized that urinary and sexual functions share common neural pathways, suggesting SNS could also modulate afferent nerves involved in sexual function (24). Limited studies have explored the use of SNS in sexual dysfunction. A metanalysis by Khunda et al. reviewed 17 studies assessing the role of SNS and sexual function in women with pelvic floor disorders (25). Sexual function was not the primary outcome in any of these studies, instead pelvic floor dysfunction and bladder dysfunction were the primary outcomes of interest. Their pooled analysis, based on data from 11 studies evaluating 573 patients before and 438 patients after SNS revealed statistically significant improvements in sexual function. Of the 11 studies, nine had SNS for urinary symptoms and two for fecal incontinence. Khunda et al. then analyzed the effect of sexual function separately in the studies, finding a positive effect in sexual function only in the nine urinary symptom studies. A secondary outcome analysis revealed SNS caused improvements in arousal, satisfaction, and pain but not orgasm or lubrication.

Persistent genital arousal disorder (PGAD), defined as the presence of recurring unwanted feelings of genital arousal or being on the verge of orgasm (genital dysthesia), is a distressing condition believed to be associated with pudendal neuropathy (26). These sensations occur without concurrent sexual thoughts or fantasies, causing significant bother and distress. Treatment options for PGAD are limited. In one series, five of six patients were improved using pudendal neuromodulation (27). Four patients completed a survey 38 months after implantation, with three of the patients reporting PNM to be the most successful treatment for their PGAD. Additionally, all four patients experienced an improvement in chronic pelvic pain and three reported improvements in bowel and bladder function.

## Peripheral nerve neuromodulation

Up to 33% of patients who undergo neuromodulation for CPP conditions have their device explanted (13). Although the precise reason for this is unknown, it could be attributed to the intricate innervation of the pelvis and elusive identification of the exact nerve target or inadequate stimulation parameters. In cases where sacral and pudendal neuromodulation have proven ineffective, peripheral nerve neuromodulation (PNNM) has emerged as a promising option. This has been made possible by use of newer wireless controlled and powered devices that do not need an implantable pulse generator (IPG) and can deliver high frequency stimulation of up to 1000 Hz resulting in a stimulation field that can result in an “electronic” nerve block. The device most commonly used are Freedom^®^ Stimulators (Curonix).

PNNM offers the advantage of providing minimally invasive pain management by targeting the exact area of pain with small devices. Personalized medicine may hold the key to addressing the needs of patients who do not respond to pudendal Interstim.

**Figure 1:**
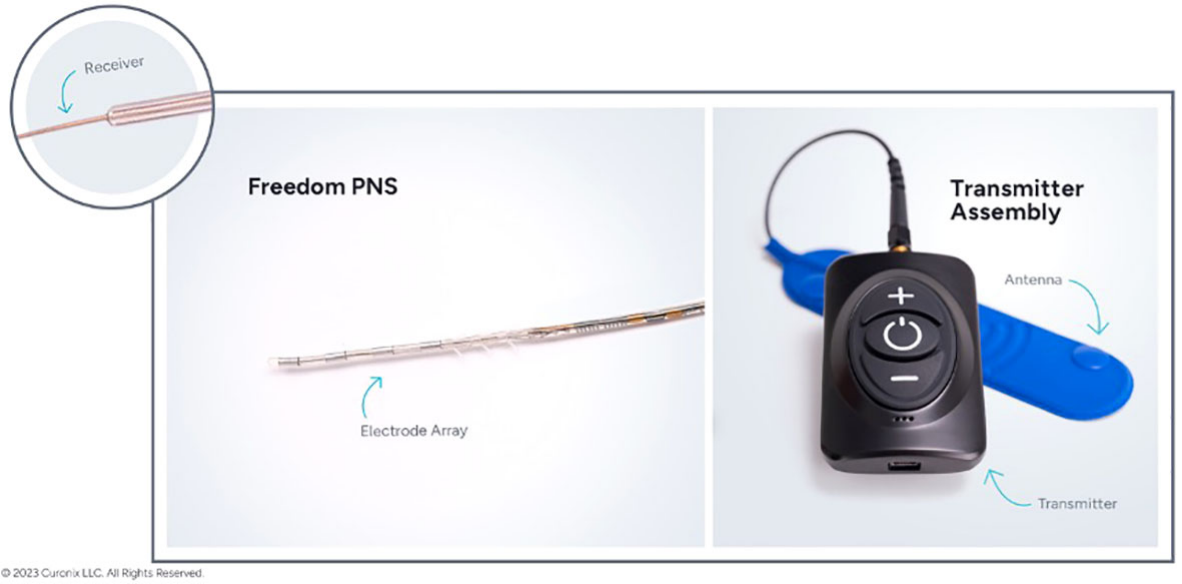
Freedom^®^ Stimulator consisting of two parts: An implanted quadripolar, tined lead (left) and an external wireless antennae and rechargeable power source called the “wearable antenna assembly (WAA)” (right). Permission for figure obtained from Curonix.

Freedom^®^ Stimulators consists of two parts: An implanted quadripolar, tined lead and an external wireless antennae and rechargeable power source called the “wearable antenna assembly (WAA)”. Pulsed radiofrequency signals are sent through the skin from the antenna to the implanted copper receiver wire. This transmits current to the ASIC computer chip integrated in the lead, igniting the chip and allowing for diverse programming options (**Figure 1**). There is no implantable battery to be replaced and a personalized medicine protocol is used for patients to determine the amount of stimulation needed per day to maintain symptom improvement. A 5–7-day trial is done with a temporary lead that is removed in the office and if patients have at least a 50% reduction in their pain levels, then they can have a permanent implant completed that can be done either unilaterally or bilaterally depending on the patient’s pain distribution (28).

Identification of the pudendal nerve during lead placement involves visualization of the anal wink and use of electromyography (EMG). Two EMG electrodes are placed in the anal sphincter and a compound muscle action potential (cMAP) is recorded. The cMAP represents the sum of all action potentials from muscle fibers stimulated, evoked by stimulation of the pudendal nerve. Presence of both the anal winc and the cMAP confirms proper pudendal nerve stimulation.

In a pilot study, Roberts et al. retrospectively reviewed the Freedom Stimulator at the pudendal nerve in patients with refractory pudendal neuralgia (29). Between September 2018 and July 2019, 13 patients (12 female and 1 male) underwent lead placement. These patients had previously failed at least one of the following treatments: medical therapy, PFPT, pudendal nerve block, trigger point injection, and prior surgery. Two (15.4%) of the patients had failed sacral Interstim, and two patients (15.4%) had failed pudendal Interstim. They found 77% of patients experienced greater than 50% improvement in pain, with 46% achieving complete pain resolution after the trial period. Follow-up was conducted by phone for seven patients at varying intervals (ranging from 22 to 759 days) after lead implantation. Of those, two patients reported marked improvement, two noted moderate improvement, and one patient reported slight improvement (greater than 50% improvement). The authors did report complications in five patients including lead migration, broken wire, and difficulties with the WAA.

Not having an implantable pulse generator allows the Freedom^®^ lead to be placed at distinct anatomical locations to manage specific pain disorders. Here are examples of PNNM used to treat post-sling pain and vulvodynia.

It is estimated that 2% of women experience pelvic pain following sling placement (30). Traditional treatment options such as topical estrogen, mesh removal, PFPT, and trigger point injections may not provide relief. Martin et al. presented a case study describing the use of the Freedom^®^ Stimulator in a patient with post-retropubic sling pain unresponsive to other treatments (31). The patient, a 50-year-old female, developed right sided pelvic pain following sling placement for stress urinary incontinence. Despite multiple surgical procedures to remove the mesh in its entirety, PFPT, and pain medications, her symptoms did not abate. Only right sided trigger point injections and a right pudendal nerve block yielded some relief, but pudendal Interstim was unsuccessful.

To address the patient’s pain, a series of retropubic blocks using triamcinolone and 0.5% ropivacaine were performed. The needle was advanced to the painful region until pain relief was noted. Based on the positive response to the retropubic block, the authors placed a Freedom stimulator in the retropubic space at the site of pain. They described specific modifications to lead placement in the retropubic space: identifying the site of pain preoperatively, bending the lead introducer to follow the curve needed to traverse the retropubic space. An incision is then made lateral to the pubic symphysis and the introducer is advanced behind the pubic bone to the finger in the vagina, at the site of pain. When the introducer was stimulated an anal wink with accompanying cMAP was observed. The authors postulated this indicated an aberrant branch of the pudendal nerve that was affected during the initial retropubic sling placement. The lead with its internal receiver was deployed at the site of the pain and activated with the WAA. At the 12-month follow-up, the patient reported over 90% improvement in her pain. She stimulates the device for 6-8 hours a day.

Vulvodynia is a challenging condition that can be managed with analgesics, PFPT and vestibulectomy. Stephens and Peters presented a case of a 63-year-old female with left vestibulodynia refractory to PFPT, excision of labial scar, and pudendal neuromodulation but responsive to PNNM (32). An 8-electrode Freedom^®^ stimulator was used to cover the entire labial surface. Specific points of pain were mapped using cutaneous stimulation in the office. These were confirmed preoperatively. An incision was made above the left labia and the electrode was tunneled along the course of pain. EMG electrodes were placed in the pelvic floor muscles, with a cMAP response seen when they were activated. The patient had almost 100% resolution of pain during the trial period, which promptly returned when the temporary lead was removed. Following placement of the permanent lead, the redundant lead was tunneled to the abdominal wall. One year postoperatively, patient reported no return of pain with daily use of the device.

## Conclusion

Neuromodulation has shown good efficacy in addressing a wide range of chronic pelvic conditions that often co-exist with pelvic pain disorders. In the treatment of urinary urgency and frequency, neuromodulation currently serves as a third or fourth-line treatment option and is FDA approved for non-obstructive urinary retention, fecal incontinence, and can be used for pelvic pain associated with urinary dysfunction. Multiple studies have shown the beneficial effects of sacral and pudendal neuromodulation in managing refractory pudendal neuralgia, a condition known for its resistance to conventional treatments. Moreover, a personalized approach of peripheral neuromodulation has opened new avenues for tailored medical interventions, extending its application to conditions such as pudendal neuralgia, post sling pain, and vulvodynia. New technologies leading to miniaturized neuromodulation devices such as Freedom^®^ stimulators (Curonix), allows us to implant leads and modulate nerves at precise pain targets. Further experience and research is needed to assess the impact of targeted neuromodulation on managing complex pelvic pain conditions.

## Author contributions

NK: Writing – original draft, Writing – review & editing. LS: Writing – review & editing. JG: Writing – review & editing. KP: Writing – review & editing.
